# Synergistic Inhibition of Wnt Pathway by HIF-1α and Osteoblast-Specific Transcription Factor Osterix (Osx) in Osteoblasts

**DOI:** 10.1371/journal.pone.0052948

**Published:** 2012-12-27

**Authors:** Dafu Chen, Yang Li, Zhiyu Zhou, Yonggang Xing, Yu Zhong, Xuenong Zou, Wei Tian, Chi Zhang

**Affiliations:** 1 Laboratory of Bone Tissue Engineering, Beijing Research Institute of Traumatology and Orthopaedics, Beijing JiShuiTan Hospital, Beijing, China; 2 Bone Research Laboratory, Texas Scottish Rite Hospital for Children, University of Texas Southwestern Medical Center, Dallas, Texas, United States of America; 3 Department of Spine, The First Affiliated Hospital of Sun Yat-sen University, Guangzhou, China; University of Alabama at Birmingham, United States of America

## Abstract

Osterix (Osx) is an osteoblast-specific transcription factor required for osteoblast differentiation. Inhibition of Wnt pathway by Osx highlights the potential for feedback control mechanisms involved in bone formation. Hypoxia-inducible factor-1α (HIF-1α) is a master regulator of hypoxia. HIF-1α has been reported to couple angiogenesis to osteogenesis. Our recent study has demonstrated that Osx and HIF-1α cooperatively regulate VEGF expression in osteoblasts. Effects of hypoxia/HIF-1α on osteoblast proliferation and related mechanisms are not well understood. In this study, osteoblast growth under hypoxia was examined. We observed that osteoblast growth was inhibited under hypoxia. To explore possible mechanisms for hypoxia/HIF-1α to inhibit osteoblast proliferation, we tested the effect of hypoxia/HIF-1α on Wnt pathway. Quantitative RT-PCR results revealed that Wnt target genes such as cyclin D1 and c-Myc were downregulated under hypoxia while HIF-1α was upregulated. Treatment of desferrioxamine, a HIF-1α activator, led to further downregulation of expressions of cyclin D1 and c-Myc in osteoblasts. On the contrary, the inhibition of HIF-1α by siRNA in osteoblasts led to the expression increase of cyclin D1 and c-Myc. These data suggest that HIF-1α inhibits Wnt pathway in osteoblasts. To examine the effect of HIF-1α on Wnt pathway, HIF-1α was cotransfected with β-catenin along with Topflash reporter in transient transfection assay. Our results showed that HIF-1α inhibited β-catenin-induced Topflash reporter activity. Interestingly, a synergistic interplay was observed between Osx and HIF-1α in the inhibition of β-catenin-induced Topflash expression. Our findings indicate that Osx and HIF-1α cooperatively inhibit Wnt pathway. This study revealed additional new information of the cooperation between HIF-1α and Osx in osteoblasts.

## Introduction

Bone formation includes two distinct processes: endochondral ossification which requires a cartilage intermediate and intramembranous ossification which forms directly from mesenchymal condensations without cartilage template. Bone formation is a highly regulated process involving the differentiation of mesenchymal stem cells to osteoblasts. Osteoblast differentiation from mesenchymal stem cells is controlled by various transcription factors and signaling proteins, including Indian Hedgehog, Runx2, Osterix (Osx), and Wnt pathway [1]. Indian Hedgehog is indispensible for endochondral ossification and the initial activation of Runx2 [2]. Runx2 is required for both endochondral and membranous ossification and needed for mesenchymal cell differentiation into preosteoblasts [3]. Osx, downstream of Runx2, is specifically expressed in osteoblasts and of low amount in prehypertrophic chondrocytes [4]. Osx was first discovered as a bone morphogenetic protein 2 (BMP-2) inducible gene in mesenchymal stem cells. *Osx* knockout mice lack bone formation, while cartilage is normal. Osx is required for osteoblast differentiation and bone formation.

The canonical Wnt pathway plays an important role in bone formation. Wnt polypeptides bind to frizzled receptors and LRP5/6 coreceptors [5]. Activation of Wnt signaling results to the accumulation and nuclear translocation of β-catenin, which then interacts with members of the Lef/Tcf family of transcription factors to activate target genes [6]. Wnt signaling is essential to osteoblast differentiation during embryonic development. Conditional inactivation of β-catenin in either skeletal progenitor cells or at a later stage of osteoblast development in mouse embryos blocks osteoblast differentiation [7], [8], [9], [10]. The Wnt signaling is also required for normal osteoblast proliferation. When β-catenin is stabilized in osteoblasts during mouse embryonic development a marked increase in osteoblast proliferation occurs [10]. Moreover *Lrp5-*null mice, which phenocopy the osteoporosis-pseudoglioma syndrome in humans [11], develop a phenotype with low bone mass because of decreased osteoblast proliferation [12]. In addition, the Wnt signaling antagonist Dkk1 prevents the activation of Wnt signaling by binding to LRP5/6. The bone formation and bone mass of heterozygous *Dkk1* mutant mice increase with an increased number of osteoblasts [13]. In contrast, the overexpression of *Dkk1* in osteoblasts leads to severe osteopenia with decreased osteoblast numbers [14]. Thus, Wnt/β-catenin signaling stimulates osteoblast proliferation. It has been reported that Osx inhibits osteoblast proliferation while it induces osteoblast differentiation [15]. The discovery that Osx inhibits the Wnt pathway highlights the potential for novel feedback control mechanisms involved in bone formation [15].

Replacing the avascular cartilage template with highly vascularized bone is the key step of endochondral ossification. During endochondral bone formation, chondrocytes model the growth plate at the long bone distal ends and become hypertrophic and hypoxic. Growth plate chondrocytes go through well-ordered and regulated phases of cell proliferation, differentiation, and apoptosis [16], [17]. Differentiation is followed by hypertrophic chondrocyte death, blood vessel invasion, and replacement of the cartilage matrix with a trabecular bone matrix. Angiogenesis and osteogenesis are coupled spatially and temporally in bone formation [18]. Blood vessel invasion from the metaphyseal region into the avascular cartilage coincides with bone formation on the cartilaginous template. The processes of endochondral bone formation and fracture repair are dependent on the blood vessel invasion [19]. Vascular endothelial growth factor (VEGF) is involved in both angiogenesis and osteogenesis. The nature of the cellular and molecular mechanisms for the transition of cartilage replacement with bone remains poorly understood. One of the driving forces is hypoxia. Hypoxia-inducible factor-1α (HIF-1α) is a master regulator of cellular response to hypoxia. For endochondral ossification, HIF-1α upregulates VEGF, and causes enhanced bone modeling [20]. Our studies have provided the first evidence that Osx directly targets VEGF expression, involving direct binding of Osx to sequence specific, GC-rich promoter elements to activate the VEGF expression in osteoblasts [21]. The observations indicate that Osx positively regulates VEGF expression while inducing osteoblast differentiation, suggesting a potential role for Osx in coordinating osteogenesis and angiogenesis. Our recent observations have demonstrated that Osx and HIF-1α cooperatively regulate VEGF expression in osteoblasts [22]. It has been speculated that the hypoxia in the chondrocytes imposes energetic limitations on the cells as they evolve from a proliferative to a terminally differentiated state [23]. However, effects of hypoxia/HIF-1α on osteoblast proliferation and related mechanisms are not well understood.

In this study, we explored the role of hypoxia/HIF-1α in osteoblast proliferation. We found that osteoblast growth was inhibited under hypoxia and that HIF-1α inhibited Wnt pathway. Interestingly, Osx and HIF-1α cooperatively inhibited Wnt pathway.

## Methods

### Plasmid constructs and Cell cultures

pEX-Osx plasmid and Topflash reporter was subcloned and used as previously described [15]. PIP2N-HIF-1α plasmid was used as previously described [22]. MC3T3 cells (ATCC) were cultured in Alpha Minimum Essential Medium with ribonucleosides, deoxyribonucleosides, 2 mM L-glutamine and 1 mM sodium pyruvate (GIBCO) and supplemented with 10% FBS and penicillin plus streptomycin. HEK293 cells (ATCC) were grown in Dulbecco's Modified Eagle Medium (GIBCO) supplemented with 10% FBS and 100 units/ml penicillin and 100 ug/ml streptomycin. Cells were cultured in 95% air/5% CO_2_ humidified incubator. Cells were trypsinized and plated before transfection.

### Hypoxia experiment and osteoblast proliferation assay

MC3T3 osteoblastic cells were maintained in Alpha Minimum Essential Medium containing 10% FBS, and cultured in normoxic (21%O_2_) or hypoxia (1%O_2_) condition incubator with 5%CO_2_ and the balanced N_2_. For osteoblast proliferation assay, MC3T3 were plated in 6-well plates at cell density of 2×10^5^ cells/well, and cultured under hypoxia for different time points from 4 hr to 72 hr before harvest and cell counting. All endpoints measured in hypoxia cells were compared with those in cells kept under normoxic condition. Desferrioxamine was purchased from Sigma (D9533-1G).

### RNA isolation and Real-time RT-PCR

Total RNA was isolated from MC3T3 osteoblasts with TRIzol reagent (Invitrogen) followed by RNeasy mini kit (Qiagen) as previously described [24]. TaqMan One-Step RT-PCR Master Mix reagent (Applied Biosystems) was used for quantitative RT-PCR. Reaction volume is 50 ul per well on 96-well plates. Analysis was performed with ABI PRISM 7500 sequence detection system (Applied Biosystems). Primers were ordered from Applied Biosystems. Transcript levels were normalized to heat shock protein 90 (HSP90) levels. All reactions were done in duplicate and all experiments were repeated at least three times. The relative mRNA expression levels were calculated according to the comparative C_T_ (ΔΔC_T_) method as described by the manufacturer (User Bulletin #2, Applied Biosystems). Target quantity is normalized to endogenous control and relative to a calibrator, and is calculated using formula: Target amount = 2^−ΔΔC^_T_.

### Protein purification and Western blot

Protein was isolated by acetone precipitation from the cell lysates as previously described [25]. The protein pellet was dissolved in 1% SDS buffer, warmed for 15 min at 55°C, and centrifuged for 5 min at 14000 rpm. Protein concentrations in the supernatant were determined using a BCA Protein Assay Kit (Pierce). Proteins were separated on 10% SDS-PAGE gels and transferred to a PVDF membrane followed by Western blot analysis. Briefly, 3% milk in TBS containing 0.1% Tween-20 was used to block non-specific binding. The blot was subsequently incubated with an anti-HIF-1α rabbit polyclonal antibody (1∶200, Abcam) or an anti-HSP90 rabbit polyclonal antibody (1∶200, Abcam) followed by a secondary antibody (peroxidase-conjugated anti-rabbit IgG 1∶5000, Sigma). After each antibody incubation, blots were extensively washed in TBS containing 0.1% Tween-20. For detection, the ECL kit (Amersham Life Sciences) was used according to the directions of the manufacturer.

### siRNA interference

MC3T3 cells were transfected by siRNA against mouse HIF-1α with Lipofectamine 2000 as previously described [26]. siRNA oligos were purchased from Thermo Scientific Dharmacon, and siGENOME Lamin A/C Control siRNA was used as a non-specific control. Cells were cultured in 6-well plates. One day before transfection, cells were plated in 1 ml of growth medium without antibiotics. Cells were 30–50% confluent at the time of transfection. For each sample, siRNA:Lipofectamine. 2000 transfection complex was prepared as follows: (1) Dilute 2 µl of 50 µM siRNA in 50 µl of Opti-MEM I Reduced Serum Medium without serum; (2) Mix Lipofectamine. 2000 gently, then dilute 3 µl in 50 µl of Opti-MEM I Medium; (3) Combine the diluted siRNA with the diluted Lipofectamine. 2000; (4) Add 100 µl of siRNA:Lipofectamine. 2000 complex to each well. After 4 hours incubation, the growth medium was replaced. Cells were cultured at 37°C in a CO_2_ incubator for 24 hours before harvest.

### Transient transfection and Topflash reporter assay

HEK293 cells were plated in 12-well tissue culture dishes and transiently transfected with 250 ng of Topflash reporters, β-catenin and expression plasmids of Osx and HIF-1α as indicated and 25 ng β-galactosidase plasmid, using FuGENE 6 reagent (Roche) according to the manufacture's instruction. After transfection, cells were incubated for 24 h before harvest. The reporter assays were analyzed with BD Monolight system (BD Biosciences). Luciferase activity was normalized by β-galactosidase activity. Values were presented as the mean ±S.D.

### Statistical Analysis

All experiments were repeated a minimum of 3 times. Data was reported as the mean ± standard deviation (S.D.). Comparisons were made between groups by Student's t test with p<0.05 being considered as statistically significant.

## Results

### Hypoxia inhibited osteoblast proliferation

**Figure 1:**
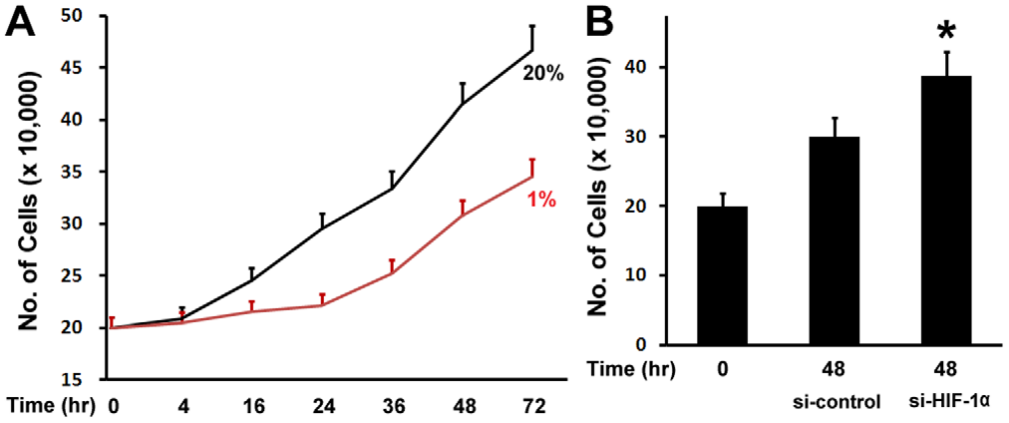
Hypoxia inhibits osteoblast proliferation. (A) Osteoblast number counts in the growth medium. MC3T3 osteoblastic cells were cultured in Alpha Minimum Essential Medium, and maintained for different time points as indicated from 4 hr to 72 hr in normoxic (20%O_2_) or hypoxia (1%O_2_) condition. (B) Inhibition of HIF-1α expression by siRNA resulted in an increase of osteoblast growth. MC3T3 osteoblastic cells were transfected by siRNA, and cultured under hypoxia for 48 hr. si-control: si-RNA control; si-HIF-1α: si-RNA against HIF-1α. A paired *t*-test was performed comparing si-control group and si-HIF-1α group. *: A star indicates statistical significance compared to control group.

To examine the effect of hypoxia on osteoblast proliferation, MC3T3 osteoblastic cells were cultured in Alpha Minimum Essential Medium, and maintained for different time points in normoxic (20%O_2_) or hypoxia (1%O_2_) condition under a humidified hypoxia incubator. We observed that MC3T3 osteoblastic cells under hypoxia condition grew slower than those in normoxia condition (Fig. 1A). Hypoxia started to inhibit cell growth at 16 hr, and the inhibition remained at 72 hr. HIF-1α is a master regulator of cellular response to hypoxia. We then asked if the inhibitory effect of hypoxia on osteoblast proliferation is related to HIF-1α. To address this question, we used siRNA technology to knockdown the expression of HIF-1α under hypoxia. Osteoblast growth was then examined under hypoxia condition. As shown in Fig. 1B, compared with osteoblast growth in si-RNA control group, inhibition of HIF-1α expression by siRNA resulted in an increase of osteoblast growth under hypoxia. These experiments therefore indicate that HIF-1α participates in hypoxia-mediated inhibition of osteoblast growth.

### Hypoxia led to downregulation of Wnt targets

**Figure 2:**
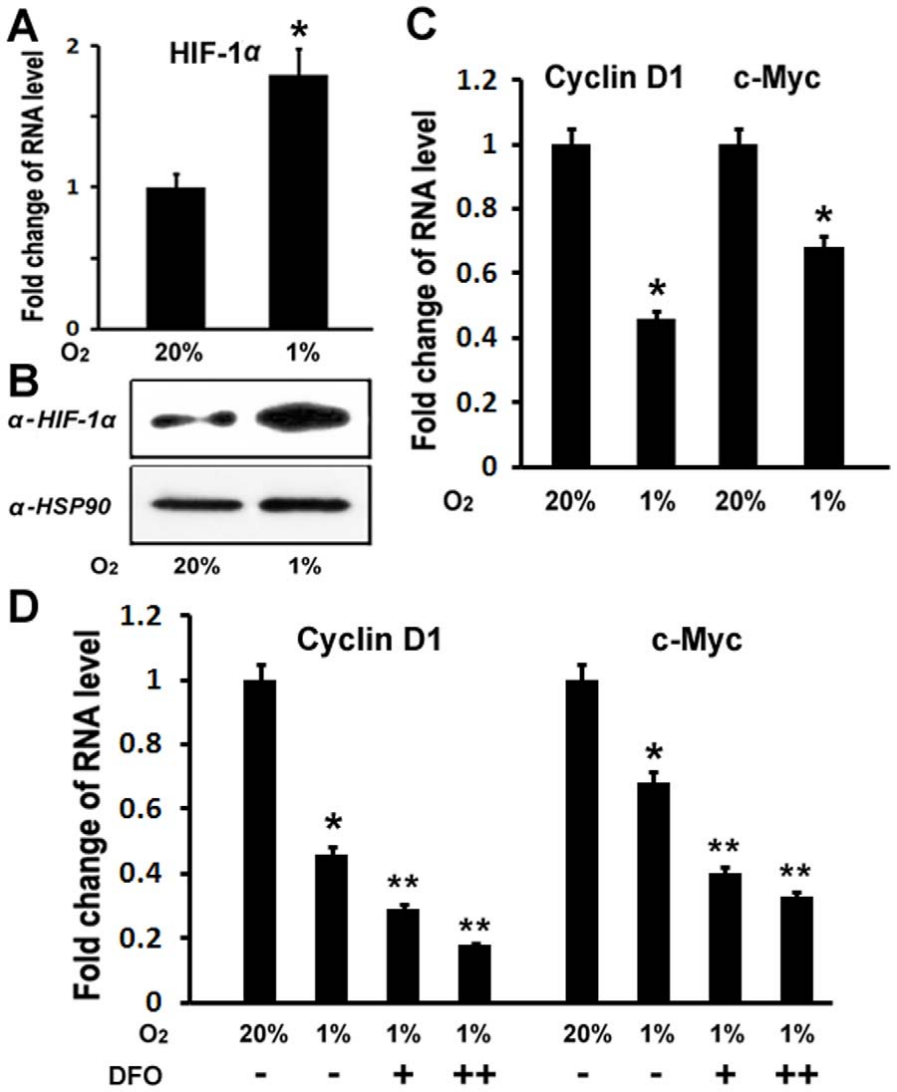
Hypoxia leads to downregulation of Wnt targets. (A) Increase of HIF-1α expression in RNA level in osteoblasts under hypoxia. RNA levels were normalized to heat shock protein 90 (HSP90). A paired *t*-test was performed comparing control group (20% O_2_) and hypoxia group (1% O_2_). *: A star indicates statistical significance compared to control group. (B) Western blotting analysis of HIF-1α expression in protein level in osteoblasts under hypoxia. Heat shock protein 90 (HSP90) was used as a loading control. (C) RNA expression levels of cyclin D1 and c-Myc as determined by quantitative real-time RT-PCR. MC3T3 osteoblasts were cultured for 48 hr under hypoxia (1%O_2_). RNA was isolated and quantitated by real-time RT-PCR. The RNA level from normoxic condition (20%O_2_) group was normalized to a value of 1. Values were presented as the mean ±S.D. A paired *t*-test was performed comparing control group (20% O_2_) and hypoxia group (1% O_2_). *: A star indicates statistical significance compared to control group. (D) RNA expression levels of cyclin D1 and c-Myc treated with DFO as determined by quantitative real-time RT-PCR. MC3T3 osteoblasts were cultured for 48 hr under hypoxia (1%O_2_), and treated with desferrioxamine (DFO). +:100 uM; ++:200 uM. The RNA level from normoxic condition (20%O_2_) group was normalized to a value of 1. Values were presented as the mean ±S.D. A paired *t*-test was performed comparing control group (20% O_2_) and hypoxia group (1% O_2_). *: A star indicates statistical significance compared to control group. A paired *t*-test was also performed comparing 1% O_2_ group and DFO group (+ and ++). **: Two stars indicate statistical significance compared to 1% O_2_ group.

To explore the possible mechanisms of hypoxia effect on osteoblast proliferation, we used quantitative real-time RT-PCR to examine the changes of gene expressions under hypoxia. It is well-known that Wnt pathway stimulates osteoblast proliferation. We asked whether hypoxia may inhibit osteoblast proliferation through inhibiting Wnt pathway. To address this question, we examined the effect of hypoxia on the expressions of Wnt target genes: Cyclin D1 and c-Myc. MC3T3 osteoblastic cells were cultured and maintained in normoxic (20%O_2_) or hypoxia (1%O_2_) condition under a humidified hypoxia incubator. Total RNA was purified 48 hr following culture in the presence or absence of hypoxia. The expressions of Cyclin D1 and c-Myc as well as HIF-1α were quantitated by real-time RT-PCR. As shown in Fig. 2A, HIF-1α RNA expression was enhanced by 1.8 fold under hypoxia compared with nomoxia, and western blotting experiments indicated that HIF-1α protein expression level also increased under hypoxia (Fig. 2B). These confirm the hypoxia-mediated upregulation of HIF-1α expression. We observed that the expressions of Cyclin D1 and c-Myc were inhibited by 54% and 32% respectively under hypoxia, compared with nomoxia (Fig. 2C). We then asked if the inhibitory effect of hypoxia on Cyclin D1 and c-Myc expressions is related to HIF-1α. To address this question, we used desferrioxamine (DFO) in this assay, a potent HIF-1α activator to increase the expression of HIF-1α under hypoxia. Cyclin D1 and c-Myc expressions were then examined under hypoxia. As shown in Fig. 2D, additions of DFO further inhibited Cyclin D1 and c-Myc expressions under hypoxia in a dose-dependent manner. These data suggest that HIF-1α is involved in hypoxia-mediated inhibition of Cyclin D1 and c-Myc expressions.

### Inhibition of HIF-1α by siRNA resulted in upregulations of Cyclin D1 and c-Myc expressions in osteoblasts

**Figure 3:**
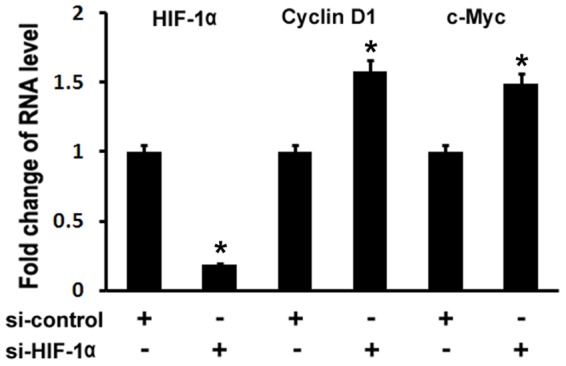
Inhibition of HIF-1α by siRNA results in upregulations of Cyclin D1 and c-Myc expressions in osteoblasts. MC3T3 osteoblasts were transfected with siRNA control or siRNA against HIF-1α. RNA was isolated 24 hr post-transfection and quantitated by quantitative real-time RT-PCR. The RNA level from the control siRNA group was normalized to a value of 1. Values were presented as the mean ±S.D. si-control: si-RNA control; si-HIF-1α: si-RNA against HIF-1α. A paired *t*-test was performed comparing si-control group and si-HIF-1α group. *: A star indicates statistical significance compared to control group.

To confirm the inhibitory of hypoxia/HIF-1α on Wnt target gene expressions, we used siRNA to knockdown HIF-1α expression in MC3T3 osteoblast cells. Real-time RT-PCR was performed to analyze Cyclin D1 and c-Myc expressions. As shown in Fig. 3, HIF-1α RNA expression was decreased by 81% using siRNA targeted against HIF-1α. Cyclin D1 RNA levels were increased by approximately 58%, and c-Myc RNA levels were increased by approximately 49%. Therefore, these loss-of-function experiments support a role for HIF-1α in inhibiting Wnt target gene expression in osteoblasts.

### HIF-1α inhibited Topflash reporter activity in a dose-dependent manner

**Figure 4:**
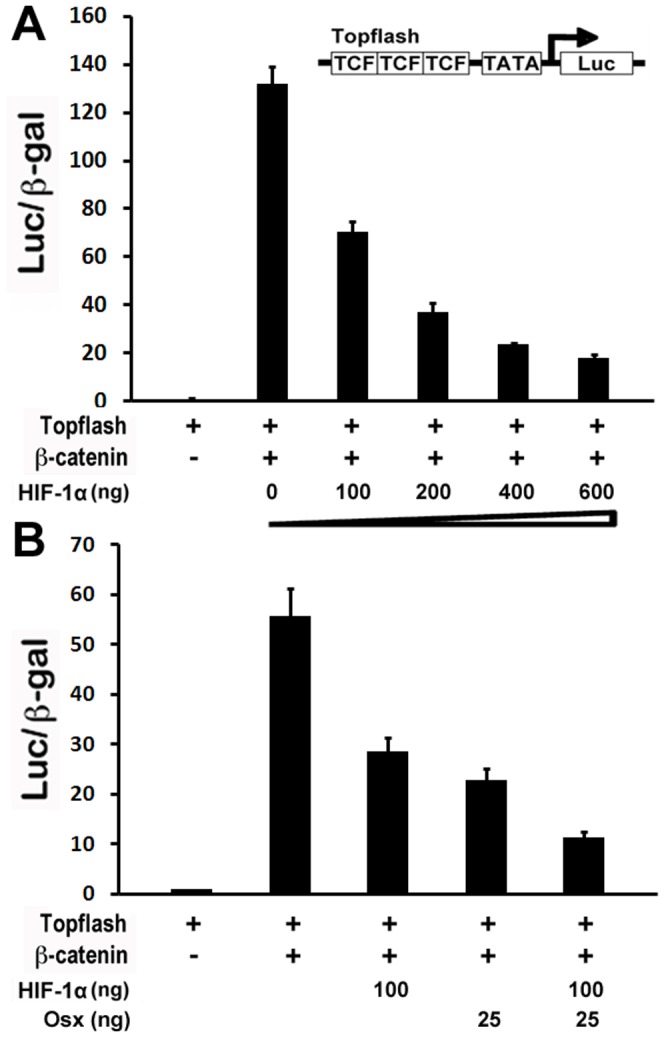
HIF-1α inhibits Wnt pathway in vitro. (A) HIF-1α inhibited Topflash reporter activity in a dose-dependent manner. HEK293 cells were transfected with a Topflash reporter along with 50 ng β-catenin without or with increasing amounts of an HIF-1α-expression plasmid as indicated. Luciferase activity was normalized by β-galactosidase activity. Values were presented as the mean ±S.D. (B) HIF-1α cooperated with Osx to inhibit Wnt pathway activity. HEK293 cells were transfected with a Topflash reporter along with 25 ng β-catenin without or with different groups of HIF-1α expression plasmid and Osx plasmid as indicated. Luciferase activity was normalized by β-galactosidase activity. Values were presented as the mean ±S.D.

We have shown that hypoxia led to downregulation of Wnt targets, and that enhanced expression of HIF-1α by DFO further inhibited Wnt target gene expressions while repression of HIF-1α by siRNA resulted in upregulation of Wnt target gene expressions. These data suggest that HIF-1α inhibits Wnt pathway in osteoblasts. Topflash reporter system is an established model in vitro as a widely used indicator of Wnt/β-catenin signal transduction [15]. To examine Wnt pathway regulation by HIF-1α, we took advantage of this established model of β-catenin-induced Topflash activation. Here, we tested the effect of HIF-1α on β-catenin-induced Topflash activation. HEK293 cells were transiently transfected with Topflash reporter and β-catenin expression vector as indicated. Expression plasmid p1p2n HIF-1α is a HIF-1α mutant which is constitutively active as previously used [27]. As shown in Fig. 4A, the β-catenin activated Topflash reporter expression as expected, and increasing amounts of HIF-1α transfection caused significantly lower expression of the Topflash reporter induced by β-catenin, indicating that HIF-1α inhibited β-catenin-induced Topflash activation in a dose-dependent manner.

### HIF-1α cooperated with Osx to inhibit Wnt pathway activity

Both Osx and HIF-1α are important for endochondral ossification during bone formation. Our recent studies have demonstrated that Osx and HIF-1α cooperatively regulate VEGF expression in osteoblasts [22]. Osx inhibits osteoblast proliferation through inhibiting Wnt pathway [15]. Here, we ask if there is any cooperation between HIF-1α and Osx to inhibit Wnt pathway. HEK293 cells were transiently cotransfected with Topflash reporter and β-catenin expression vector along with Osx expression plasmid. As shown in Fig. 4B, transfection of 100 ng HIF-1α or 25 ng Osx alone inhibited β-catenin-induced Topflash reporter expression by 49% and 59%, respectively. Interestingly, cotransfection of such amounts of HIF-1α and Osx resulted in a further inhibition of β-catenin-induced Topflash by 80%. These data indicate that there is a synergistic interplay between HIF-1α and Osx in Wnt pathway inhibition.

## Discussion

Osx is an osteoblast-specific transcription factor that regulates the expression of essential genes needed for appropriate osteoblast differentiation and bone formation. The discovery that Osx inhibits the Wnt pathway highlights the potential for novel feedback control mechanisms involved in bone formation [15]. Our recent report has demonstrated that Osx and HIF-1α cooperatively regulate VEGF expression in osteoblasts [22]. In this study, we examined the role of hypoxia/HIF-1α in osteoblast proliferation and Wnt pathway. The findings presented here indicate that hypoxia/HIF-1α inhibits osteoblast proliferation and that HIF-1α cooperates with Osx to inhibit Wnt pathway.

First, we showed that hypoxia inhibited osteoblast proliferation. This was supported by cell proliferation assay. MC3T3 osteoblastic cells grew slower under hypoxia than those in normoxia condition (Fig. 1A). HIF-1α is the crucial mediator of the adaptive response of cells to hypoxia. The oxygen dependent degradation of HIF-1α is controlled by a family of HIF prolyl hydroxylases. Under normoxic conditions, HIF-1α is hydroxylated by prolyl hydroxylases that act as oxygen sensors. Hydroxylation of specific proline residues on HIF-1α is followed by proteasomal degradation. Under hypoxic conditions, HIF-1α is stabilized, translocated to the nucleus, and forms a heterodimer with HIF-1β to regulate target genes. These target genes are involved in a variety of cellular processes including angiogenesis, energy metabolism, cell proliferation and survival, vasomotor control, and matrix metabolism [28]. To address whether HIF-1α is involved in hypoxia-mediated inhibition of osteoblast proliferation, siRNA technology was used to knockdown the expression of HIF-1α in this study. Fig. 1B demonstrated that inhibition of HIF-1α expression by siRNA led to an increase of osteoblast growth compared with osteoblast growth in si-RNA control group. These experiments indicate that HIF-1α participates in hypoxia-mediated inhibition of osteoblast proliferation.

The current study also addresses possible mechanisms for hypoxia/HIF-1α to inhibit osteoblast proliferation. As a crucial mediator of hypoxia, HIF-1α is ubiquitous [29]. It is controversial how HIF-1α affects cell proliferation. Studies performed in cell lines or in ES cell-derived tumors indicate that HIF-1α can modulate tumor cell growth by controlling both metabolic functions and expression of angiogenic growth factors such as VEGF [30], [31]. Despite an initial report showing that HIF-1α would act as a negative factor for the growth of ES cell-derived tumors, some studies support the model that the lack of HIF-1α inhibits tumor growth [31], [32]. In this study, we demonstrated that hypoxia/HIF-1α inhibited osteoblast proliferation. We also investigated mechanisms that could mediate inhibitory action of hypoxia/HIF-1α in osteoblast proliferation. HIF-1 is a heterodimer that consists of HIF-1α, the oxygen sensitive subunit, and the constitutively expressed HIF-1β. HIF-1 activates target gene transcription by binding to the hypoxia-responsive elements in the proximal promoter region of the oxygen responsive genes. This study indicates that Wnt pathway is one of possible mechanisms for hypoxia/HIF-1α to inhibit osteoblast proliferation. This is supported by several evidences: 1) RT-PCR results revealed that Wnt target genes such as cyclin D1 and c-Myc were downregulated under hypoxia; 2) the treatment of HIF-1α activator DFO further downregulated expressions of cyclin D1 and c-Myc; 3) the inhibition of HIF-1α by siRNA in osteoblasts led to the expression increase of cyclin D1 and c-Myc; 4) our transfection assay showed that HIF-1α inhibited β-catenin-induced Topflash reporter activity. However, our study cannot rule out other possible mechanisms of the effect of hypoxia/HIF-1α on osteoblast proliferation, such as hypoxia-induced PH value change, some other hypoxia-related factors, like vascular endothelial growth factor, insulin-like growth factor II, and transforming growth factor β1, etc. It has been reported that Osx inhibits osteoblast proliferation while it induces osteoblast differentiation [15]. We showed in that study that Osx negatively regulated β-catenin-induced Topflash activation in a dose-dependent manner. Since our recent studies indicate that Osx and HIF-1α collaboratively control VEGF expression in osteoblasts [22], it is interesting to explore the possibility whether Osx and HIF-1α may work together to control Wnt pathway. Indeed, our current results indicated that Osx and HIF-1α inhibited Wnt pathway in a synergistic manner (Fig. 4B). Because of the role of Wnt pathway in stimulating osteoblast proliferation, we speculate that hypoxia-induced inhibition of osteoblast proliferation may be at least partially through inhibition of Wnt pathway by HIF-1α.

In summary, we present here that hypoxia/HIF-1α inhibit osteoblast proliferation, and that HIF-1α has a synergistic effect with Osx on the inhibition of Wnt pathway. While additional studies need to address the role of synergistic inhibition of Wnt pathway by HIF-1a and Osx, these early stage studies revealed additional new information of the cooperation between HIF-1α and Osx in osteoblasts.
